# *Bcl-2* Overexpression Improves Survival and Efficacy of Neural Stem Cell-Mediated Enzyme Prodrug Therapy

**DOI:** 10.1155/2018/7047496

**Published:** 2018-06-20

**Authors:** Rachael Mooney, Asma Abdul Majid, Daniel Mota, Adam He, Soraya Aramburo, Linda Flores, Jennifer Covello-Batalla, Diana Machado, Joanna Gonzaga, Karen S. Aboody

**Affiliations:** Department of Developmental and Stem Cell Biology, Beckman Research Institute of City of Hope, 1500 East Duarte Road, Duarte, CA 91010, USA

## Abstract

Tumor-tropic neural stem cells (NSCs) can be engineered to localize gene therapies to invasive brain tumors. However, like other stem cell-based therapies, survival of therapeutic NSCs after transplantation is currently suboptimal. One approach to prolonging cell survival is to transiently overexpress an antiapoptotic protein within the cells prior to transplantation. Here, we investigate the utility and safety of this approach using a clinically tested, *v*-*myc* immortalized, human NSC line engineered to contain the suicide gene, cytosine deaminase (CD-NSCs). We demonstrate that both adenoviral- and minicircle-driven expression of the antiapoptotic protein *Bcl-2* can partially rescue CD-NSCs from transplant-associated insults. We further demonstrate that the improved CD-NSC survival afforded by transient *Bcl-2* overexpression results in decreased tumor burden in an orthotopic xenograft glioma mouse model following administrations of intracerebral CD-NSCs and systemic prodrug. Importantly, no evidence of CD-NSC transformation was observed upon transient overexpression of *Bcl-2*. This research highlights a critical need to develop clinically relevant strategies to improve survival of therapeutic stem cell posttransplantation. We demonstrate for the first time in this disease setting that improving CD-NSC survival using *Bcl-2* overexpression can significantly improve therapeutic outcomes.

## 1. Introduction

Tumor-tropic neural stem cells (NSCs) have been engineered to localize a variety of therapeutic agents to invasive brain tumors [1], with NSC-mediated enzyme prodrug treatment strategy being the first to be tested clinically. In 2013, we completed a safety/feasibility study (NCT01172964) in which a v-*myc* immortalized NSC line was modified to express *Escheria coli* cytosine deaminase (CD; HB1.F3.CD21; CD-NSCs). This enzyme converts an inactive prodrug, 5-fluorocytosine (5-FC), to the chemotherapeutic effector 5-fluorouracil (5-FU) [2]. Patients with recurrent high-grade glioma received injections of CD-NSCs into the peritumoral resection or biopsy site at the time of surgery, followed by 7 days of oral 5-FC. Results from 15 patients with 1 treatment cycle demonstrated safety (of up to 50 million CD-NSCs), nonimmunogenicity, brain tumor-localized prodrug conversion, and evidence of NSC migration to distant tumor sites [3]. Two-phased 1 dose-escalation enzyme prodrug gene therapy trials for recurrent glioma patients are now underway. The first involves intracerebral CD-NSC administration (up to 150 million NSCs) followed by oral 5-FC (NCT02015819). The second involves intracerebral administration of the CD-NSC line further engineered to secrete a modified human carboxylesterase (hCE1m6) [4], which converts the prodrug irinotecan (CPT-11) to the more potent topoisomerase-1 inhibitor, SN-38 [5] (NCT02192359). Patients receive repeat treatment cycles via a Rickham catheter placed at the time of resection on biopsy. These first-in-human trials are exciting, because tumor-tropic NSCs are postulated to migrate to invasive tumor foci that typically elude effective distribution by traditional enzyme expression vectors.

However, one of the overlooked challenges that may be limiting the therapeutic potential of cell-mediated therapies is suboptimal cell survival posttransplantation. In the case of CD-NSC enzyme prodrug therapy, the apoptotic stimuli encountered upon administration into the tumor resection cavity are significant and unavoidable. Thawed cells are placed into a stressful environment that contains poor matrix support and high concentrations of reactive oxygen species [6]. Studies of NSC transplantation into the brain for neurological disorders report primary NSC survival of <4–10% within the first few days [7]. This suggests that posttransplantation survival of the genetically modified CD-NSC line is a critical parameter to investigate for optimal therapeutic efficacy.

Hostile transplant environments are a challenge for many cell therapies. One emerging strategy to address this problem is to overexpress the antiapoptotic gene, B-cell lymphoma 2 *(Bcl-2)*. *Bcl-2* is a mild protooncogene that, if *transiently* overexpressed, could protect donor cells from apoptotic stimuli during the critical 1-week window in which prodrugs are administered [2] and when apoptosis is commonly observed after transplantation [8]. In fact, the mechanism by which the CD/5-FC therapy induces apoptosis converges on *Bcl-2* modulation [9], so this approach is ideally suited for this particular NSC-mediated enzyme prodrug therapy. Thus far, transient *Bcl-2* overexpression has effectively improved posttransplantation viability and efficacy of embryonic stem cells [10] and mesenchymal stem cells used to treat ischemic cardiac insults [11] and skeletal defects [12], respectively. Transient *Bcl-2* overexpression has been accomplished using traditional nonintegrating adenoviral vectors or using minicircle technology, which lacks any potentially inflammatory viral and/or bacterial sequences [12].

Here, we investigate transient *Bcl-2* overexpression as an effective, safe approach to achieve prolonged posttransplantation survival of CD-NSCs. One main concern is the potential risk of neoplastic transformation of implanted NSCs given that *Bcl-2* overexpression is associated with tumors of both lymphoid and epithelial origin [13–15]. It is accepted that *Bcl-2* expression is insufficient to induce uncontrolled cell proliferation without cooperation from a second oncogene [16, 17]. However, when *stably* coexpressed, *myc* and *Bcl-2* can cooperate to increase tumor incidence in a variety of lymphoid and epithelial cell types as well as transgenic models [18, 19]. Because CD-NSCs were immortalized using *v-myc*, it is necessary to ensure that transient *Bcl-2* expression does not transform CD-NSCs.

We hypothesize that transformation of CD-NSCs will not occur upon transient *Bcl-2* overexpression based on the following rationale. First, *Bcl-2* overexpression would only transiently occur during the short (<1 week) window in which NSC survival is critical for maximum prodrug conversion and tumor tropism. Second, *v*-*myc* expression within CD-NSCs undergoes constitutive downregulation upon transplantation, perhaps through developmental and epigenetic mechanisms that suppress endogenous cellular *myc* in NSCs during mitotic arrest [20]. Finally, even if a small fraction of injected CD-NSCs maintains coexpression of v-*myc* and *Bcl-2*, they would quickly succumb to the antiproliferative chemotherapeutics generated upon administering prodrug intended for the dividing tumor cells.

The studies presented here confirm that while CD-NSCs are certainly susceptible to oxidative stress and anoikis, transient *Bcl-2* overexpression partially rescues them. Importantly, we observed no evidence that *Bcl-2* overexpression impairs tumor tropism and prodrug expression or induces neoplastic transformation. We also demonstrate that *Bcl-2* overexpression improves both CD-NSC survival and the therapeutic efficacy observed after one round of treatment.

## 2. Results

### 2.1. *Bcl-2* Expression Constructs

**Figure 1:**
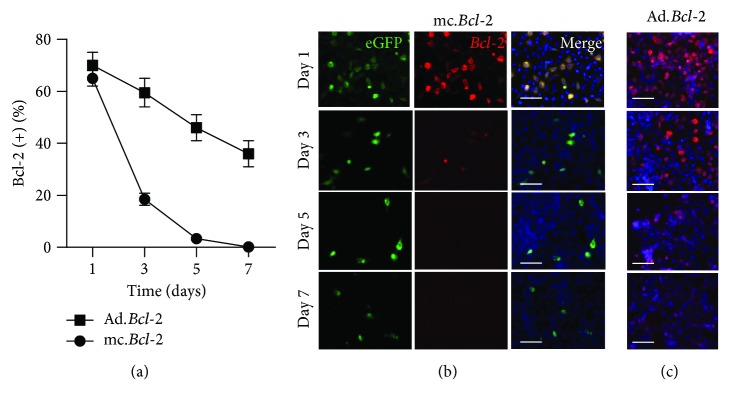
Time course of transient *Bcl-2* expression in NSCs. (a) Flow cytometric quantification of *Bcl-2* positive cells over 1 week posttransfection with minicircle constructs (circle) or posttransduction with adenovirus (square). (b-c) Representative immunofluorescent images demonstrating a decline in eGFP and *Bcl-2* expression in both minicircle-transfected (b) and adenovirally transduced NSCs (c) over 1 week. Scale bars in both (b) and (c) = 50 *μ*m.

Both adenoviral and minicircle expression vectors were utilized to transiently overexpress *Bcl-2* within CD-NSCs. After establishing optimal adenoviral transduction parameters (Supplementary Figure 1), initial *Bcl-2* overexpression was observed in 66 ± 4% of CD-NSCs as assessed by flow cytometric analysis of *Bcl-2* positive cells (Figures 1(a) and 1(c)). A nontarget (firefly luciferase expressing) adenoviral vector was used as control vector in all studies. A comparable level of initial *Bcl-2* overexpression (65 ± 5% of CD-NSCs) was achieved using our eGFP-labelled minicircle construct (Figures 1(a) and 1(b)). As controls, two other minicircle constructs were generated containing a *Bcl-2* shRNA or a nontargeting scrambled sequence (Supplementary Figure 2). Because the initial transfection efficiency of both control constructs was lower (<20% of CD-NSCs), these controls were used only during select *in vitro* studies.

### 2.2. *Bcl-2* Expression Efficiency and Time Course Assessments

While the percentage of NSCs initially overexpressing *Bcl-2* was comparable between the adenoviral and minicircle expression constructs, the rate at which they lost *Bcl-2* expression differed greatly. The adenoviral construct resulted in a gradual decline in the percentage of *Bcl-2* positive NSCs, with up to 40% of CD-NSCs *Bcl-2 (+)* by day 7 (Figures 1(a) and 1(c)). In contrast, the percentage of *Bcl-2* (+) CD-NSCs quickly declined after transfection with our *Bcl-2* minicircle construct, dropping to 20% (+) by day 3 and no *Bcl-2 (+)* cells by day 7 (Figures 1(a) and 1(c)). This decline in *Bcl-2* expression occurred more rapidly than the decline in eGFP (+) CD-NSCs, suggesting a short duration of *Bcl-2* expression even in minicircle-transfected CD-NSCs. Thus, both adenoviral and minicircle expression vectors achieved transient *Bcl-2* expression. Both constructs were pursued further because the optimal duration for *Bcl-2 over*expression is still not clear with respect to affording increased CD-NSC survival without compromising safety.

### 2.3. *BCL-2* Expression Improves HB1.F3.CD NSC Survival *In Vitro*

**Figure 2:**
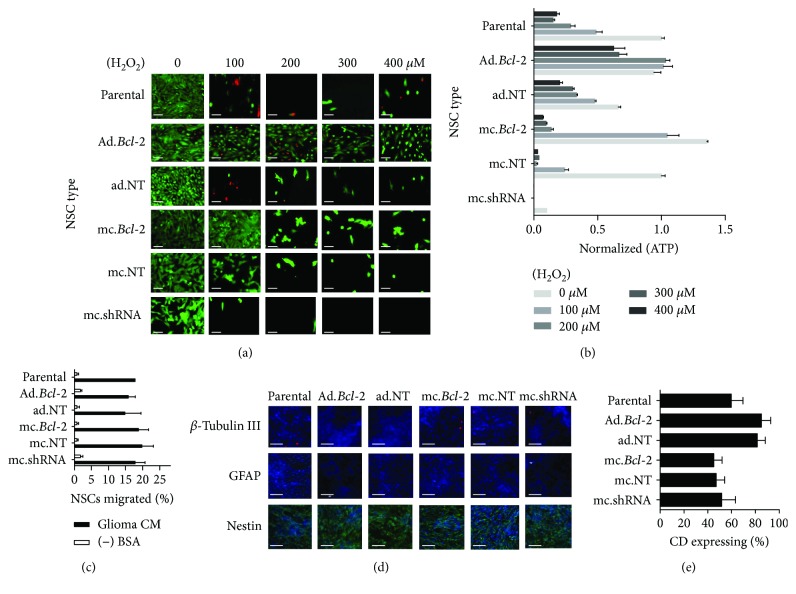
Bioactive effects of *Bcl-2* expression in NSCs. (a) Representative fluorescent images of NSC cultures after a 96 hr incubation in increasing H_2_O_2_ doses. Live and dead cells were, respectively, visualized with calcein-AM (green) and ethidium bromide (red). (b) Absolute ATP content present in culture was quantified and normalized with respect to values observed when parental NSCs were cultured with no exogenous H_2_O_2._ (c) Tumor tropism of parental and transduced/transfected NSCs in response to U87 glioma-conditioned media measured *in vitro*. Data are expressed as percentage of migrated cells, where 100% is 1 × 10^5^. (d) Representative parental and transduced/transfected NSC cultures immunostained for lineage-specific protein. (e) Flow cytometry analysis of *E. coli* cytosine deaminase expression parental and transduced/transfected NSCS immunostained with anti-CD.

We next tested if *Bcl-2* overexpression confers CD-NSCs with a survival advantage under controlled *in vitro* insults. First, cultured parental and *Bcl-2*-overexpressing CD-NSCs were exposed to increasing H_2_O_2_ doses to mimic the high-oxidative stress present upon transplantation into the brain. NSC viability was assessed both qualitatively (LIVE/DEAD imaging) and quantitatively (total cellular ATP measurements). LIVE/DEAD images show a significant loss in viable (green) parental CD-NSCs (Figure 2(a), first row) after four days of exposure to even 100 *μ*M H_2_O_2_, demonstrating this cell line is clearly susceptible to oxidative stress-induced damage. Similar results were observed when NSCs were modified with either the nontargeted adenovirus (Figure 2(a), third row) or the nontarget minicircle vector (Figure 2(a), fifth row). Slightly increased susceptibility to oxidative stress was apparent when NSCs were first transfected with the minicircle containing *Bcl-2* shRNA (Figure 2(a), sixth row). In contrast, the cultures containing *Bcl-2*-overexpressing NSCs contained an increased number of viable CD-NSCs after exposure to 100 *μ*M H_2_O_2_ (Figure 2(a); adenovirus, second row; minicircle, fourth row). In fact, adenovirus-driven *Bcl-2* expression resulted in an increased number of viable CD-NSCs even after being exposed to 400 *μ*M H_2_O_2_ for four days.

Quantitative ATP measurements yielded results consistent with the LIVE/DEAD imaging. Increasing H_2_O_2_ concentrations resulted in significantly reduced total ATP levels when applied to parental CD-NSCs (Figure 2(b)). Transducing the CD-NSCs with the control adenovirus did not rescue declining ATP levels; however, transducing with the *Bcl-2* transgene was effective in maintaining total ATP at levels equal to that observed when NSCs were not exposed to H_2_O_2_ (Figure 2(b)). A similar, though less potent effect was observed when NSCs were transfected with minicircles containing the *Bcl-2* transgene. In this case, the rescued ATP levels were observed only at H_2_O_2_ concentrations of 100 *μ*M. In addition, total ATP levels in culture were significantly reduced when NSCs were transfected with minicircles containing *Bcl-2* shRNA, even when NSCs were not exposed to H_2_O_2_, an effect not observed using a scrambled shRNA sequence (Figure 2(b)).

Furthermore, *Bcl-2* overexpression resulted in no significant changes in the identity or therapeutic function of CD-NSCs. The NSCs remained immunopositive for the neural stem cell marker, nestin, and immunonegative for neuronal (*β*-tubulin III) and glial (GFAP) lineage markers (Figure 2(d)). *Bcl-2* overexpression had no detrimental effect on NSC tropism to tumor-conditioned media (Figure 2(c)) and no significant effect on the expression of the prodrug-converting enzymes, CD (Figure 2(e)), or carboxylesterases (Supplementary Figure 3).

### 2.4. *BCL-2* Expression Improves HB1.F3.CD NSC Survival *In Vivo*

**Figure 3:**
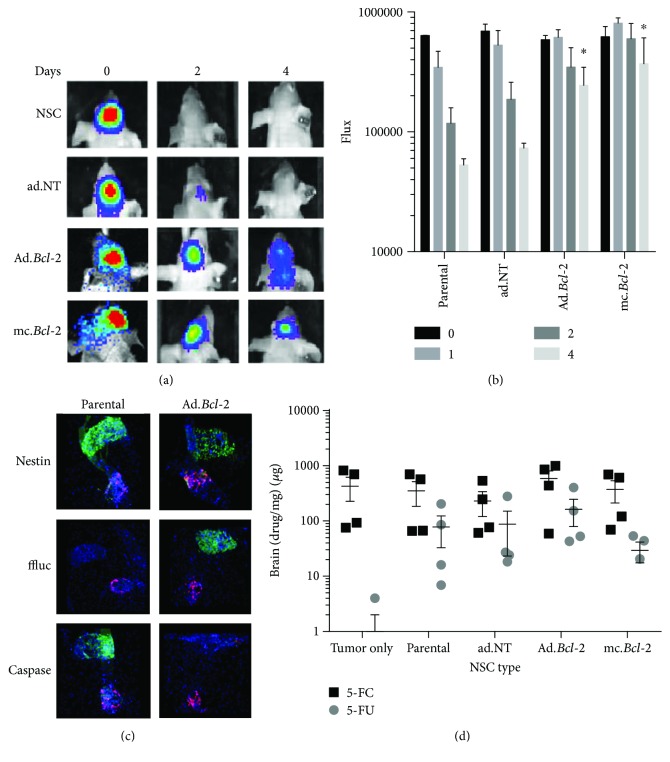
Bioactive effects of *Bcl-2* expression in NSCs *in vivo*. (a–c) NSC survival after ipsilateral transplantation into glioma-inoculated mice. (a) Representative xenogen images of ffluc-expressing NSCs at select time points. (b) Quantification of xenogen flux in all animals (mean + SEM) shown at select time points. Asterisks indicate statistically significant increases in NSC flux relative to time-matched parental NSCs (*p* < 0.05). (c) Representative tissue sections showing DsRed.U251 tumor (red) and NSC (green) injection sites 1 day after NSC injections. NSCs are stained for nestin (present in live and dead NSCs), eGFP (expressed only in live NSCs), and active caspase-3 (expressed only in dying NSCs). (d) LC/MS/MS quantification of 5-FC prodrug (black) and 5-FU (gray) active drug present in mice brains implanted with both parental and genetically modified NSCs.

To determine if transient *Bcl-2* overexpression can extend the time CD-NSCs remain viable *in vivo*, genetically modified and parental firefly luciferase- (ffluc-) expressing CD-NSCs were injected ipsilateral to preestablished U251.DsRed glioma orthotopic xenografts. Then, longitudinal bioluminescence imaging was used to monitor the duration of NSC-derived ffluc expression (Figures 3(a) and 3(b)). Despite a robust ffluc signal in all groups on the day of implantation, both the parental CD-NSCs and the CD-NSCs transduced with control adenovirus had minimal signal remaining two days later (Figure 3(a), top two rows). In contrast, CD-NSCs modified to express *Bcl-2* using either the adenoviral or minicircle vectors exhibited prolonged ffluc signal that was still visible 4 days later (Figure 3(a), bottom two rows). Thus, NSCs exhibit prolonged viability when engineered to transiently overexpress *Bcl-2*, a result that becomes statistically significant by day 4 (*p* < 0.05) (Figure 3(b)).

To confirm that the *BCL-2*-overexpressing CD-NSCs are in fact more viable than the parental NSCs, three brains from both the parental and Ad.*Bcl-2* group were harvested one day after implantation to immunologically assess the extent of CD-NSC survival. Representative fluorescence microscopic images of serially sectioned brains show a nestin (+) CD-NSC injection site (green) located near the Ds. Red U251 tumor (Figure 3(c), top row). Adjacent sections were stained for both active caspase-3/7 as a marker of apoptotic cells (Figure 3(c), bottom row) and ffluc as a marker for viable CD-NSCs (Figure 3(c), middle row). These images demonstrate that the vast majority of parental CD-NSCs are brightly positive for active caspase-3/7 and largely negative for ffluc, suggesting they were no longer viable enough to produce this protein (intracellular degradation rate of firefly luciferase = 3 hrs [21]). In contrast, Ad.*Bcl-2* CD-NSCs demonstrated intense ffluc staining but negligible active caspase-3/7 staining.

### 2.5. Therapeutic Advantage of *Bcl-2* Modified HB1.F3.CD NSC *In Vivo*

**Figure 4:**
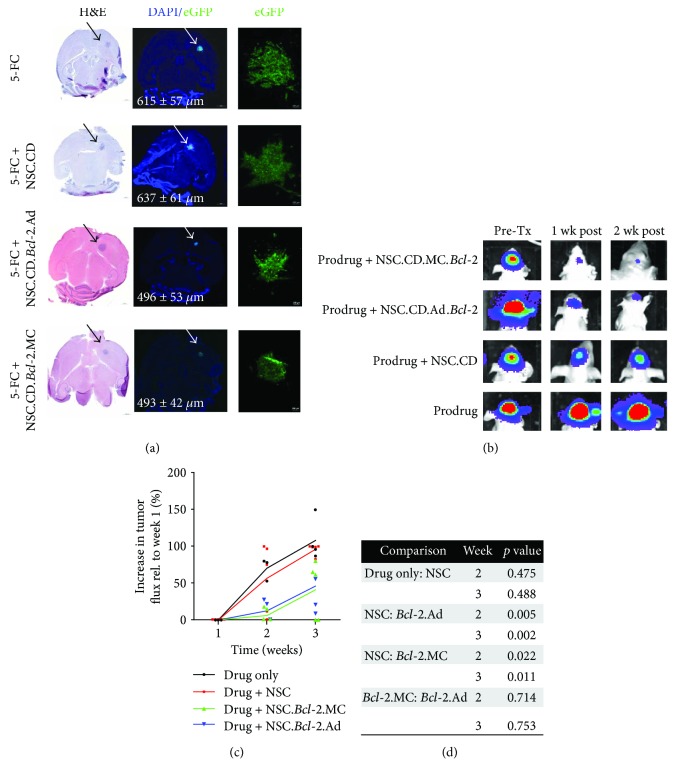
Therapeutic advantage of *Bcl-2* expression in NSCs. (a) Representative brain sections showing the relative sizes of U251.eGFP.ffluc tumor 3 weeks after coimplantation with either parental or transduced/transfected NSC.CDs. All groups received 500 mg/kg of the prodrug 5-FC. Brain slices with the maximal tumor surface area are shown stained with H&E (left panel) and DAPI (center panel). In the fluorescent DAPI images, eGFP + tumor cells are visible (green). Arrows indicate tumor location. Average maximum tumor diameter is provided in white text. (b) Representative bioluminescent images of ffluc-expressing U251 glioma cells at select time points after coimplantation with either parental or transduced/transfected NSC.CDs. (c) Quantification of bioluminescent tumor flux in all animals (mean + individual datapoints shown). (d) Bioluminescent imaging *p* value table showing MC.*Bcl-2* and Ad.*Bcl-2* treatment groups achieved a statistically significant reduction in tumor bioluminescent flux signal.

Interestingly, the improved CD-NSC survival did not translate to increased conversion of the 5-FC prodrug into the active effector, 5-FU (Figure 3(d)) as assessed in brains tumors harvested two days after NSC administration. Nonetheless, we utilized longitudinal bioluminescence imaging to monitor any changes in the progression of U251.eGFP.ffluc glioma orthotopic xenografts if treated with either parental or *Bcl-2*-expressing CD-NSCs, followed by 5 consecutive days of prodrug administration (Figures 4(b) and 4(c)). In this experiment, the tumor cells were coinjected with CD-NSCs to ensure consistent intratumoral biodistribution in each experimental group. As expected, tumor progression was observed when mice received only the 5-FC prodrug. When NSCs were present to convert the prodrug into 5-FU, a noticeable qualitative decrease in tumor flux was observed 1 week after treatment (Figure 4(b)).

The decrease in tumor flux seemed to be more substantial in mice that received *Bcl-2*-expressing NSCs, so we calculated the average % increase in luminescence flux that occurred in each treatment group. The results at week 3 demonstrate a significant delay in tumor progression observed when NSCs are modified to overexpress *Bcl-2* using either the adenoviral or the minicircle vector (Figure 4(c)). The relative tumor burdens present within brains harvested at this 3-week time point are consistent with the noninvasive imaging results, with visibly smaller tumors present in mice that received *Bcl-2*-expressing NSCs (Figure 4(a)). A similar result was observed in mice that contained preestablished, patient-derived glioma cells, where the decrease in tumor flux in NSC-treated mice was more substantial when NSCs were modified to overexpress *Bcl-2* (Supplementary Figure 4).

### 2.6. *Bcl-2*-Expressing HB1.F3.CD NSCs Are Nontumorigenic

**Figure 5:**
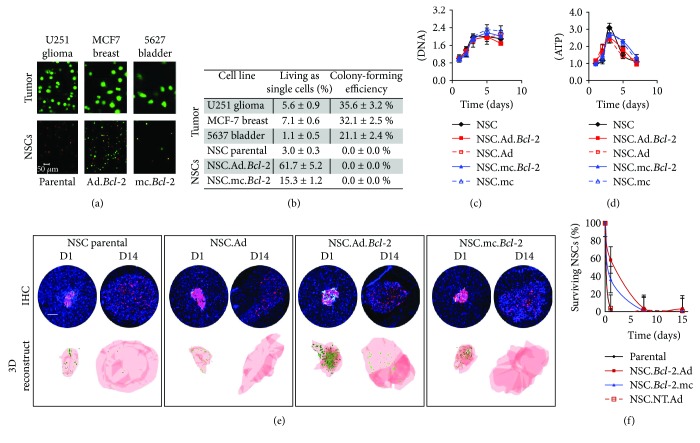
Tumorigenicity of *Bcl-2* modified HB1.F3.CD NSCs. (a-b) *Bcl-2*-expressing NSCs showed no tendency to form tumorigenic colonies characteristic of cancer cells when cultured for 7 days in agar. (a) Representative 20x confocal microscopy z-stacks obtained on day 7 after calcein-AM labeling. Tumor colonies are visible in the top panel (ordered right to left: U251 glioma, MCF7 breast cancer, and 5637 bladder cancer) and NSCs are visible in the bottom panel (ordered right to left: parental, Ad.*Bcl-2*, and mc.*Bcl-2*). Scale bar in lower left image = 50 *μ*m which applies to all images in Figure 5(a). (b) Table displaying quantitative data obtained using ImageJ analysis software to count and size colonies in 5 images per condition. Values are normalized with respect to initial day 0 seeding counts. Colonies were defined as clusters with diameters > 20 *μ*m. (c-d) Total DNA (c) and ATP (d) levels measured as NSCs proliferated in monolayer culture over the course of 1 week. (e-f) Decline of eGFP positive NSCs over the course of two weeks after implantation into tumor- and nontumor-bearing brains. (e) Representative brain sections and 3D renderings of tumors reconstructed from serially sectioned brains highlighting the distribution of eGFP-expressing NSCs within a DsRed-expressing glioma. Scale bar = 100 *μ*m. (f) Quantified estimate of the percent of injected eGFP-expressing NSCs present within tumors over the course of 2 weeks.

Having demonstrated that *Bcl-2*-expressing CD-NSCs show prolonged viability, it was important to confirm that the *Bcl-2* modification did not transform CD-NSCs into tumor-initiating cells. As a preliminary *in vitro* assessment, CD-NSCs were cultured in nonadherent agarose cultures known to induce anoikis in normal but not cancerous cells. Results show that while established tumorigenic cell lines were able to overcome a lack of integrin signaling and form proliferative colonies, neither parental nor *Bcl-2*-expressing CD-NSCs formed colonies (Figures 5(a) and 5(b)). *Bcl-2* overexpression did, however, significantly increase the percent of NSCs that remained viable as isolated single cells (Figures 5(a) and 5(b)). Eventually, however, even the *Bcl-2*-expressing NSCs died as evidenced by a sharp decline in intracellular ATP levels over 72 h of culture to reach negligible levels (data not shown). Total DNA and ATP were also measured within growing CD-NSC cultures to confirm that *Bcl-2*-expressing NSCs did not exhibit increased proliferation rates and still exhibit contact-inhibited growth patterns indistinguishable from that of parental NSCs (Figures 5(c) and 5(d)).

We also confirmed that *Bcl-2* overexpression does not result in abnormal CD-NSC proliferation *in vivo*. In this experiment, the glioma line expressed DsRed, and the NSCs expressed eGFP, but no prodrugs were administered. Brains were harvested on day 1, 7, and 14 to monitor the extent of CD-NSC proliferation when initially coinjected with DsRed glioma cells (Figures 5(e) and 5(f)). We observed that the *Bcl-2* overexpression, particularly using the adenoviral expression vector, significantly improved the percentage of eGFP-expressing CD-NSCs still present on day 1 (60% versus 4% of parental CD-NSCs). However, the acute rejection and/or apoptosis that occurred over the subsequent 2 weeks [22] effectively eliminated most of the transplanted CD-NSCs in all groups (Figures 5(e) and 5(f)). By day 7, NSCs in all groups had ceased dividing as confirmed by negative PCNA and Ki-67 staining (data not shown).

Finally, a pilot long-term tumorigenicity assessment was performed using Bcl*-2*-expressing CD-NSCs. We injected up to 10 times the clinically relevant human cell dose (1.0 × 10^6^) into the brains of nontumor-bearing immunodeficient mice. All mice had normal gait, appetite, alertness, hydration, neurological symptoms, and weight during the week after NSC injection and through time to planned euthanasia. Two months later, brain sections were examined for the presence of viable and/or proliferative NSCs. Hematoxylin and eosin (H&E) histochemistry of tissue sections through the brain appeared normal in all mice. Focal gliosis and hemosiderin-laden macrophages were slightly discernable at NSC injection sites; however, no immunoreactive NSCs remained (data not shown).

## 3. Discussion

NSCs offer a unique taxis-based cell delivery vehicle that can actively target anticancer therapeutics to invasive tumors like glioma. As is the case with most other stem cell therapies, the promise of NSC-mediated antiglioma therapy is currently hampered by suboptimal stem cell survival after transplantation. Here, our data show that transient overexpression of *Bcl-2* by human CD-NSCs can increase their survival posttransplantation, which translates to improved therapeutic efficacy in an orthotopic xenotransplantation glioma model. These results are consistent with a growing body of work demonstrating that *Bcl-2* overexpression can safely enhance the survival of other types of therapeutic stem cells including hematopoeitc stem cells [23], adipose-derived mesenchymal stem cells [12], and peripheral NSCs [24].

To transiently overexpress *Bcl-2* within CD-NSCs, we tried two different nonintegrating approaches: adenoviral transduction and minicircle transfection. Both approaches are clinically relevant. If pursuing the adenoviral vector, it will be necessary to carefully evaluate the immunogenic potential and confirm the replication-deficient status of intracellular adenovirus particles [25]. We have obtained approval for use of adenovirus-driven rCE expression in our ongoing CD-NSC.hCE1m6 recurrent glioma trial (NCT02192359) [5]. Our head-to-head comparison found the adenoviral approach resulted in a longer duration in which a significant percentage of NSCs overexpressed *Bcl-2*. The quick decline in *Bcl-2*-expressing NSCs after minicircle transfection was surprising given that an expression duration of 7–14 days is more typically reported for minicircle expression vectors [26]. Perhaps, duration of minicircle-driven *Bcl-2* expression may be extended if all remnant bacterial DNA was eliminated.

The major challenges of cell survival *in vivo* include an ischemic tissue environment, immune cell recognition, loss of ECM, and oxidative stress. Here, we demonstrate that *Bcl-2* expression improves the ability of NSCs to survive amidst two of these insults *in vit*ro. We show that *Bcl-2* overexpression improves CD-NSC survival under anoikis-inducing conditions within agarose gel, without inducing colony formation indicative of transformed cells. We also demonstrate that, consistent with previous reports using other cell types [27, 28], *Bcl-2* overexpression protects NSCs from the oxidative stress induced upon exposure to hydrogen peroxide. Furthermore, we report no changes in proliferation rate, therapeutic enzyme expression, tropic ability, or differentiation status of CD-NSCs upon overexpression of *Bcl-2*. While there have been previous reports that *Bcl-2* overexpression may increase neuronal differentiation from E11.5 peripheral rat neural precursor cells [29], this was not observed for CD-NSCs. In contrast to primary NSCs, which differentiate in response to a myriad of external cues, the v-*myc* immortalization of CD-NSCs seems to stabilize their undifferentiated status. *Bcl-2*-expressing CD-NSCs survived longer than native CD-NSCs when transplanted into the brains of immunodeficient mice, as evidenced by prolonged firefly luciferase signal and significantly reduced active caspase-3 staining. We also demonstrate that improved CD-NSC survival translates to an improved therapeutic advantage *in vivo.* While the detected drug conversion levels did not increase, the tumor-derived luciferase signal and postharvest tumor volume measurements suggested delayed tumor progression when treated with *Bcl-2*-expressing NSCs.

As expected, we found no evidence that transient *Bcl-2* expression transforms NSCs into tumor-initiating cells using either minicircle technology or adenoviral transduction. Though both vectors are categorized as nonintegrating, we still need to be conscious of their transformative potential if a *Bcl-2* transgene were to hypothetically integrate into this v-*myc* immortalized CD-NSC line. Harui and coworkers demonstrated that the frequency of adenovirus integration into chromosomal DNA was around 10^−3^/–10^−5^ events per cell [30]. Estimates of episomal/plasmid integration rates are similar (10^−4^) [31]. This implies that when implanting a clinical dose of 150 million CD-NSCs into patients [3], up to 150 NSCs may contain integrated DNA using either of these approaches. Thus, for cell therapies utilizing transient *Bcl-2* overexpression to improve cell survival clinically, it may be prudent to incorporate a suicide gene as well. The results of this study suggest that the potential therapeutic benefit afforded by *Bcl-2*-mediated cell survival could outweigh the risks.

## 4. Conclusion

Tumor cells frequently take advantage of proteins within the apoptotic pathway to overcome a myriad of insults. We can learn from nature and manipulate this pathway within therapeutic donor cells in our efforts to combat the tumor. We provide evidence that transient overexpression of the antiapoptotic protein, *Bcl-2*, within human CD-NSCs improves their resistance to transplant-associated insults. Their improved survival also translates to improved therapeutic outcomes in a xenograft orthotopic mouse model. Importantly, we observed no evidence that transient *Bcl-2* overexpression transformed CD-NSCs, suggesting this approach is safe enough to merit further study. Together, these data highlight the importance of developing strategies that improve the survival of therapeutic NSCs and other cell-based therapies. These strategies will be critical to ongoing efforts to achieve improved therapeutic outcomes for glioma patients receiving NSC-mediated prodrug conversion therapies, as well as other stem cell treatments of CNS diseases.

## 5. Materials and Methods

### 5.1. Cell Culture

All cell lines were cultured in Dulbecco's Modified Eagle's Medium (DMEM) (Invitrogen) supplemented with 10% fetal bovine serum (Gemini Bio), 1% l-glutamine (Invitrogen), and 1% penicillin/streptomycin (Invitrogen) and maintained at 37°C in a humidified incubator (Thermo Electron Corporation) containing 6% CO_2_. When cell reached 80% confluency, they were passaged using a 0.25% trypsin/EDTA solution (Invitrogen); media were changed every 2-3 days.

#### 5.1.1. Glioma Cell Lines

Firefly luciferase-expressing U251 and PBT-017 (U251.ffluc, PBT-017.ffluc) and DsRed.U251 were provided by Dr. Christine Brown. U87 human glioma cell lines were obtained from the American Type Culture Collection. U87 cells were used to generate tumor cell-conditioned media by replacing culture media with serum-free media when cells were 80% confluent, followed by a 48-hour incubation

#### 5.1.2. Neural Stem Cell Lines

The human, v*-myc* immortalized, HB1.F3 NSC line was obtained from Dr. Seung Kim (University of British Columbia) [32]. Extensive characterization studies have demonstrated the HB1.F3 line is chromosomally and functionally stable, nontumorigenic, and minimally immunogenic (HLA class II negative [1, 33]). This cell line was further transduced with lentivirus to stably express either eGFP [20] and firefly luciferase [34] and used to track stem cell distribution *in vivo*.

### 5.2. *Bcl-2* Expression Constructs

#### 5.2.1. Adenoviral Transduction

Adenovirus vectors for *Bcl-2* with cytomegalovirus promoters were purchased from Vector Biolabs. For NSC transduction, NSCs were plated in 6-well plates at 90% confluence without penicillin/streptomycin. The following day, culture media was replaced with transduction media containing no penicillin/streptomycin, 10% FBS, 2 *μ*g/ml protamine-sulfate (Sigma-Aldrich), and viral particles at a multiplicity of infection of 20 which was determined empirically to result in the greatest number of *Bcl-2* positive cells after 24 hours (Supplementary Figure 1). After 24 hours, the transduction media was removed, and complete transduction efficiency confirmed using flow cytometry and immunohistochemistry (Supplementary Figure 1). The percent cytotoxicity following transfection was determined as 100 × (number of nontransfected adherent NSCs – number of adherent transfected NSCs)/(number of nontransfected adherent NSCs). This is an indicator of cell viability following transfection. Each transduction was carried out in triplicate and repeated at least 2 times.

#### 5.2.2. Minicircles

*(1) Construct Generation*. Commercially available minicircle vector backbones in the MC-easy minicircle production kit (System Bioscience) were used to generate eGFP.*Bcl-2*, *Bcl-2*.shRNA, and NT.shRNA minicircle constructs. The vector contains a multiple cloning sites and poly(A) tail flanked by attP and attB sites for PhiC31 integrase recombination and 32x Scel sites for bacterial backbone degradation, which is kanamycin resistant.

*(2) Overexpressing Minicircles*. The eGFP.*Bcl-2* gene insert was excised from a commercially available plasmid pEMD-*Bcl-2* (EMD Biosciences) using Nhe1 and EcoR1 restriction sites. The purified insert was subcloned into a linearized pMC.CMV.MCS-EF1-GFP-SV40PolyA minicircle parental plasmid using the multiple cloning sites. The pMC_*Bcl-2*.eGFP plasmid was purified and transformed into ZYCY10P3S2T *E. coli*. Minicircles were generated as per manufacturer's instructions. Minicircle and insert size were verified by performing electrophoresis on diagnostic restriction enzyme digests.

*(3) Knockdown Minicircles*. The short hairpin shRNA inserts were purchased from Invitrogen (*Bcl-2* fwd: 5′-GAT CCA ACA TCG CCC TGT GGA TGA CTT TCA AGA GAA GTC ATC CAC AGG GCG ATG TTT TTT TG-3′; *Bcl-2* rev: 5′-GTT GTA GCG GGA CAC CTA CTG AAA GTT CTC TTC AGT AGG TGT CCC GCT ACA AAA AAA CTT A-3^″^; Nontarget: Fwd: 5′- GAT CCA ATT CTC CGA ACG TGT CAC GTT TCA AGA GAA CGT GAC ACG TTC GGA GAA TTT TTT TG-3′; rev: 5′- GTT AAG AGG CTT GCA CAG TGC AAA GTT CTC TTG CAC TGT GCA AGC CTC TTA AAA AAA CTT A-3′). These inserts were used to generate *Bcl-2*.shRNA and NT.shRNA minicircles.

*(4) Minicircle Transfection*. Before transfection, 9.0 × 10^5^ NSCs were seeded into individual wells of 6-well plates. After a 24-hour incubation in growth medium without penicillin/streptomycin, the cells were exposed to DNA-Lipofectamine LTX complexes that each contained 2.5 *μ*g of minicircle plasmid DNA/well of cells. DNA-Lipofectamine LTX complexes were made by first diluting plasmid DNA and Lipofectamine LTX (Invitrogen, Carlsbad, CA, USA) in two independent 125 *μ*l volumes of Opti-MEM medium (Invitrogen) without serum and mixed gently. After a 5 min incubation with the Plus Reagent at room temperature, the DNA and Lipofectamine LTX in Opti-MEM were combined and incubated for an additional 5 min at room temperature to allow the DNA-Lipofectamine LTX complexes to form. The DNA-Lipofectamine LTX complexes were then added to each well containing cells and medium. The vol/wt ratios of Lipofectamine LTX/DNA are shown in Supplementary Figure 2. The cells were incubated in transfection media for an additional 24 hours before efficiency analysis. Each transfection was carried out in triplicate and repeated at least 2 times.

### 5.3. Bcl-2 Expression Efficiency and Time Course Assessments

#### 5.3.1. Flow Cytometry

At select timepoints, transfected cells were resuspended in PBS before analyzing on a GuavaCyte Flow Cytometer (GuavaCyte). Transduced cells were fixed and permeabilized (Fix & Perm Cell Permeabilization kit, Invitrogen, GAS 003) and incubated 40 min with Anti-*Bcl-2* (cat number 138800, Invitrogen) then 20 min with goat anti-mouse Alexa-488 before flow cytometric analysis. Transfection efficiency was determined as the number of positive NSCs/total NSCs. Histograms were generated using FlowJo (Tree Star, Ashland, OR, USA).

#### 5.3.2. Immunohistochemistry

Standard immunological techniques were employed. Briefly, plated cells were rinsed and fixed with 4% paraformaldehyde prior to blocking for 1 hour with immunoblot. Primary antibody was applied overnight at 4°C, then after rinsing, goat anti-mouse Alexa-546 was applied. After a 4-hour incubation, the cells were rinsed, stained with DAPI (Thermo Fisher), and mounted with fluorescence mounting medium (DAKO). Cultures were imaged using a Nikon Eclipse TE2000-U microscope equipped with a SPOT RT Slider digital camera (Diagnostic Instruments). Primary antibody omission served as negative controls, and no immunoreactivity was observed.

### 5.4. Bioactive Effects of *Bcl-2*-Modified HB1.F3.CD NSC *In Vitro*

#### 5.4.1. In Vitro Insult Assays

Parental and transduced/transfected NSCs were cultured for 96 hours under increasing doses of H_2_O_2._ Resulting viability was assessed qualitatively using LIVE/DEAD kit (Life Technologies) and quantitatively measuring absolute ATP present in culture using CellTiter-Glo Luminescent Cell Viability Assay (Promega) according to the manufacturer's instructions.

#### 5.4.2. Tumor Tropism

Modified Boyden chamber chemotaxis assays were performed using 24-well cell culture plates with polycarbonate inserts (pore diameter, 8 *μ*m) (Millipore, Billerica, MA, USA) as described previously [35]. Conditioned media from U87 glioma and 5% BSA/DMEM were added to the lower chamber of wells (500 *μ*l/well, triplicate samples). Inserts were placed into wells, and suspensions of parental or transfected/transduced NSCs were added to the upper chamber (1 × 10^5^ cells/250 *μ*l suspended in 5% BSA/DMEM to each well). After incubation (4 h, 37°C), cells that did not migrate were removed from the inner surface of the filter. The membrane tray was then placed in a new lower chamber containing prewarmed Accutase (Sigma-Aldrich) for 10 min at 37°C. Detached cells in the buffer were then transferred to a V-bottom 96-well plate and centrifuged (1500 rpm, 5 min). The buffer was aspirated, and cells were lysed with cell lysis buffer. The absolute amount of DNA present in 1 × 10^5^ NSCs and the cells that migrated were quantified using PicoGreen reagent according to the manufacturer's instructions. Plot shows mean ± SEM is shown (3 experiments; *n* = 9 samples).

#### 5.4.3. Differentiation

After four days of culture, cells were fixed in 4% paraformaldehyde, then standard immunocytochemical techniques were used to visualize cell-type-specific protein expression using the following primary antibodies: nestin (MAB 5326, Millipore), *β*-tubulin (PRB-435, Covance), and GFAP (AB5804, Millipore).

#### 5.4.4. Prodrug-Converting Enzyme Expression

*(1) Cytosine Deaminase*. One day after transfection/transduction, NSCs were fixed and permeabilized and incubated 40 min with Anti-CD (BD Pharmingen, 557,862) then 20 min with goat anti-mouse FITC (BD Pharmingen, 55598) before flow cytometric analysis. Histograms were generated using FlowJo (Tree Star, Ashland, OR, USA).

*(2) Carboxylesterase*. One day after transduction, CE enzyme activity was measured by conversion of *o*-nitrophenyl acetate substrate to *o*-nitrophenol and determined by spectrophotometry at 420 nm as previously described [36].

### 5.5. Bioactive Effects of *Bcl-2*-Modified HB1.F3.CD NSC *In Vivo*

#### 5.5.1. Tumor Xenograft Model

Athymic nude mice were anesthetized with an intraperitoneal injection of 132 mg/kg ketamine and 8.8 mg/kg xylazine. Mice were then immobilized in a stereotactic apparatus and received stereotactically guided intracranial injections of cell suspension 2 mm lateral, 0.5 mm anterior to bregma, tracked from a depth of 2.5 mm to 2.25 mm to 2.0 mm; 0.667 *μ*l of cell suspension was injected at each level, 2 *μ*l total. Injections were performed with a 30-gauge 5 *μ*l Hamilton syringe over 3–5 minutes. Two minutes elapsed before moving to the next injection level to minimize backflow through the needle track. After retracting the needle, bone wax was used to occlude the burr hole, and skin was closed with skin glue. To establish glioma xenografts, mice received U251.DsRed human glioma cells (5 × 10^5^ cells) at 1 week prior to NSC.eGFP.ffluc injections. In tumor-inoculated brains, intracranial injections contained 5 × 10^5^ of parental or transduced/transfected NSCs injected into either the ipsilateral or contralateral hemisphere. Buprenorphine analgesic (0.05 mg/kg) was administered subcutaneously to relieve postoperative pain. Results were obtained from 3 independent experiments that resulted in 6 mice per group. All animal protocols were approved by the City of Hope Institutional Animal Care and Use Committee. Mice were euthanized consistent with the recommendations of the Panel of Euthanasia of the American Veterinary Medical Association when they appeared to be in discomfort or distress as judged by independent animal care personnel. Mice were housed in an AAALAC-accredited facility and were given food and water ad libitum.

#### 5.5.2. Xenogen Imaging

For a period of 4 days, firefly luciferase-expressing NSCs were imaged in mice using a charge-coupled device camera (Xenogen IVIS-100) coupled to the Living Image acquisition and analysis software. Mice were anesthetized with isoflurane gas then received an intraperitoneal injection of D-luciferin substrate (suspended in PBS at 4.29 mg/mouse). Images were captured while the mice were anesthetized by isoflurane (1.5 L/oxygen, 4% isoflurane) and kept in an induction chamber. Light emission was measured over an integration time of 5 minutes at 12 min after injection of luciferin. To account for baseline differences across animals, each animal's recordings were standardized to the signal measured at day 0. Cell survival curves were generated using standardized data. A drop in flux intensity was interpreted as cell death, and this was confirmed with immunostaining.

#### 5.5.3. Immunological Tissue Analysis

Mice were harvested by CO_2_ asphyxiation, then brains were removed and fixed by immersion in 4% paraformaldehyde for 24 h before sinking in 30% sucrose for 48 h. The tissues were frozen in Tissue Tek OCT (Sakura Finetek Europe B.V.) and sectioned sagittally on a cryostat (Leica 17–20). Sections (10 *μ*m thick) were collected on positively charged slides (Thermo Fisher) for immunocytochemistry. Standard immunocytochemical techniques were used to visualize cell viability using the following primary antibodies: nestin (MAB 5326, EMD Millipore), eGFP (ab2980, Abcam), and active caspase-3 (AB3623, EMD Millipore).

#### 5.5.4. 5-FC to 5-FU Conversion In Vivo

Two days after NSC injections, mice were administered intraperitoneal 5-FC (500 mg/kg) (Sigma). 2 hours later (at the peak of 5-FU conversion), brains were harvested and quartered. The quarter containing the tumor/NSC injection was used to determine concentrations of 5-FC and 5-FU by liquid chromatography-tandem mass spectrometry LC-MS/MS. LC-MS/MS analysis was performed using a Waters Acquity HPLC system (Waters Corp.) with a Waters Quattro Premier XE Mass Spectrometer. High-performance liquid chromatography (HPLC) separation was achieved using a Synergi Hydro-RP 4 *μ*m 150 × 2.0 mm analytical column (Phenomenex) preceded by a Phenomenex C_18_ guard column. The column temperature was maintained at 30°C, and the flow rate was 0.4 ml/minute. The mobile phase consisted of A (20 mM ammonium acetate buffer, pH 3.5) and B (acetonitrile). The following gradient program was used: 20% B (hold, 0–3 minutes), 20%–68% B (3–6 minutes), 68% B (hold, 6–6.2 minutes), 68%–20% B (6.2–6.3 minutes), 20% B (hold, 6.3–8 minutes). The total run time was 8 minutes. The electrospray ionization source of the mass spectrometer was operated in positive ion mode with a cone gas flow of 80 liters/hour and a desolvation gas flow of 700 liters/hour. The capillary voltage was set to 0.6 kV, and the cone and collision cell voltages were optimized to 60 V and 36 eV for CPT-11, 48 V and 26 eV for SN-38, and 45 V and 23 eV for camptothecin (internal standard). The source temperature was 125°C, and the desolvation temperature was 450°C. A solvent delay program was used from 0 to 4.7 minutes and from 6.1 to 8 minutes to minimize the mobile phase flow to the source. MassLynx (Waters Corp.) version 4.1 software was used for data acquisition and processing. Positive electrospray ionization of 5-FC and 5-FU produced abundant protonated molecular ions (MH+) at m/z 587.31, 393.21, and 349.15, respectively. Fragmentation of these compounds was induced under collision-induced dissociation conditions and acidic mobile phase. The precursor → product ion combinations at m/z 587.31 → 124.14 for 5-FU and 393.21 → 349.20 for 5-FC were used in multiple-reaction monitoring mode for quantitation. Under optimized assay conditions, the retention times for 5-FC and 5-FU were 5.25 and 5.62 minutes, respectively.

### 5.6. Therapeutic Advantage of *Bcl-2*-Modified HB1.F3.CD NSC *In Vivo*

#### 5.6.1. Tumor Xenograft Model

The same xenograft model described in Section 5.5.1 was utilized with the following modifications: (1) U251.eGFP.ffluc was used instead of U251.DsRed, (2) dil-labeled NSCs were used instead of NSC.eGFP.ffluc, and (3) a mixture of 2e5 NSCs and 2e5 tumor cells was coinjected instead of injecting the NSCs 1 week after tumor implantation.

#### 5.6.2. Treatment Schedule

Mice were administered 500 mg/kg 5-FC intraperitoneally 2 days after NSC tumor injections, and 5 mice per group were harvested for LC/MS/MS analysis of prodrug conversions (see Section 5.5.4). On days 5–9, mice received daily 500 mg/kg 5-FC administrations 2 days after surgery. Control mice were similarly injected with 1x PBS only.

#### 5.6.3. Xenogen Imaging

Firefly luciferase-expressing tumor cells were imaged weekly for 3 weeks as described in Section 5.5.2. To account for baseline differences across animals, each animal's recordings were standardized to the signal measured at day 0. A gain in flux intensity was interpreted as tumor cell growth, and this was confirmed with immunostaining.

#### 5.6.4. Immunological Tissue Analysis

Three weeks after implantation, all brains were harvested and sectioned to visualize tumor volume as described above (see Sectio5.5.3). Standard immunocytochemical techniques were used to visualize tumor size and NSC distribution.

### 5.7. Tumorigenicity of *Bcl-2*-Modified HB1.F3.CD NSCs

#### 5.7.1. In Vitro Colony Formation Assay

A standard soft agar colony-formation assay was used to assess cellular anchorage-independent growth *in vitro*. Human tumor cells (U251 glioma, 5637 bladder, and MCF7 breast, ATCC) and HB1.F3.CD NSCs (parental and *Bcl-2* expressing) were encapsulated at 1^5^ cells/ml within 50 *μ*l, 1% *w*/*w* agarose hydrogels cured in a 96-well plate. On day 0 or after culturing for 7 days in complete growth media, gels were incubated with Calcein-AM and ethidium bromide (Life Technologies) to visualize live and dead cells, respectively, then imaged using a confocal microscope (Zeiss). ImageJ software was used to count and size cells present in z-stacks compiled from 13 optical slices spaced 100 *μ*m apart.

#### 5.7.2. In Vitro Proliferation

NSC proliferation rates *in vitro* were determined by quantifying total DNA levels present in culture using the PicoGreen assay (Invitrogen) as per manufacturer's instructions. Total ATP levels were also quantified with the CellTiter-Glo Luminescent Cell Viability Assay (Promega). Data was obtained from 2 separate experiments involving 4-5 cultures per time point.

#### 5.7.3. In Vivo Tumorigenicity

The same xenograft models described in Sections 5.5.1 and 5.6.1 were utilized. Brains were harvested 0, 1, 10, 21, and 60 days after NSC transplantation then sectioned as described in Section 5.5.3. Standard immunoctyochemical techniques were used to visualize the number of proliferative NSCs using a PCNA primary antibody (MAB242, Chemicon). In addition, the tumorigenicity of *Bcl-2*-expressing NSCs was assessed by implanting 1 × 10^6^ ffluc.*Bcl-2* NSCs into the brains of 3 nude mice lacking any tumor burden.

### 5.8. Statistical Analysis

Data are presented as mean ± SEM unless otherwise stated. Statistical significance was determined using a two-tailed Student's *t*-test (^∗^ *p* < 0.05) unless otherwise stated.

## Supplementary Materials

Supplementary Materials Additional flow cytometry and viability quantification are provided showing the percent of NSCs that are Bcl-2 (+) after adenoviral transduction at increasing multiplicities of infection (Supplementary Figure 1). We further provide maps and electrophoretic confirmation of minicircle construct generation and flow cytometric quantification of the percent of NSCs that are eGFP (+) and Bcl-2 (+) (Supplementary Figure 2). We also provide evidence that Ad.*Bcl-2* NSCs do not exhibit impaired activity of the prodrug-converting enzyme, carboxylesterase (Supplementary Figure 3). Finally, we provide additional support that Bcl-2-mediated enhancements in posttransplantation NSC viability translated to improved antitumor efficacy in a patient-derived glioma model (Supplementary Figure 4).
