# Supplementary material for: Bcl-2 Overexpression Improves Survival and Efficacy of Neural Stem Cell-Mediated Enzyme Prodrug Therapy
Source: Stem Cells Int. 2018 Jun 20;2018:7047496. doi: 10.1155/2018/7047496 (PMC6031202; doi:10.1155/2018/7047496)
Supplement: Supplementary Materials — Additional flow cytometry and viability quantification are provided showing the percent of NSCs that are Bcl-2 (+) after adenoviral transduction at increasing multiplicities of infection (Supplementary Figure 1). We further provide maps and electrophoretic confirmation of minicircle construct generation and flow cytometric quantification of the percent of NSCs that are eGFP (+) and Bcl-2 (+) (Supplementary Figure 2). We also provide evidence that Ad.Bcl-2 NSCs do not exhibit impaired activity of the prodrug-converting enzyme, carboxylesterase (Supplementary Figure 3). Finally, we provide additional support that Bcl-2-mediated enhancements in posttransplantation NSC viability translated to improved antitumor efficacy in a patient-derived glioma model (Supplementary Figure 4). [file 7047496.f1.docx]

**Supplementary Figures:** *Bcl-2* overexpression improves survival and efficacy of neural stem cell-mediated enzyme prodrug therapy

Rachael Mooney^1*^, Asma Abdul Majid^1^, Daniel Mota^1^, Adam He^1^, Soraya Aramburo^1^, Linda Flores^1^, Jennifer Covello-Batalla^1^, Diana Machado^1^, Joanna Gonzaga^1^, Karen S. Aboody^1,3*‡^


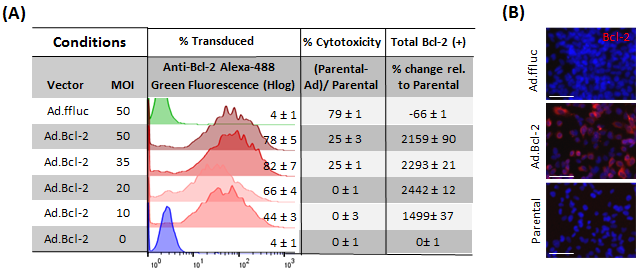


**Supplementary Figure 1: Adenoviral-driven *Bcl-2* expression in NSCs.** a) NSCs were seeded at 50% confluency and allowed 48 hrs for CAR expression to recover. NSCs were then adenovirally transduced at various MOIs (panel 1) for 24 hours in the presence of 1µg/mL protamine sulfate before utilizing flow cytometry to assess transduction efficiency (panel 2) and cytotoxicity (panel 3). The total number of NSCs overexpressing *Bcl-2* was then calculated (panel 4). b) Representative fluorescent images demonstrating positive *Bcl-2* expression in cells transduced (MOI = 20), but not in parental or vector control cultures. Scale bar = 50µm.

**
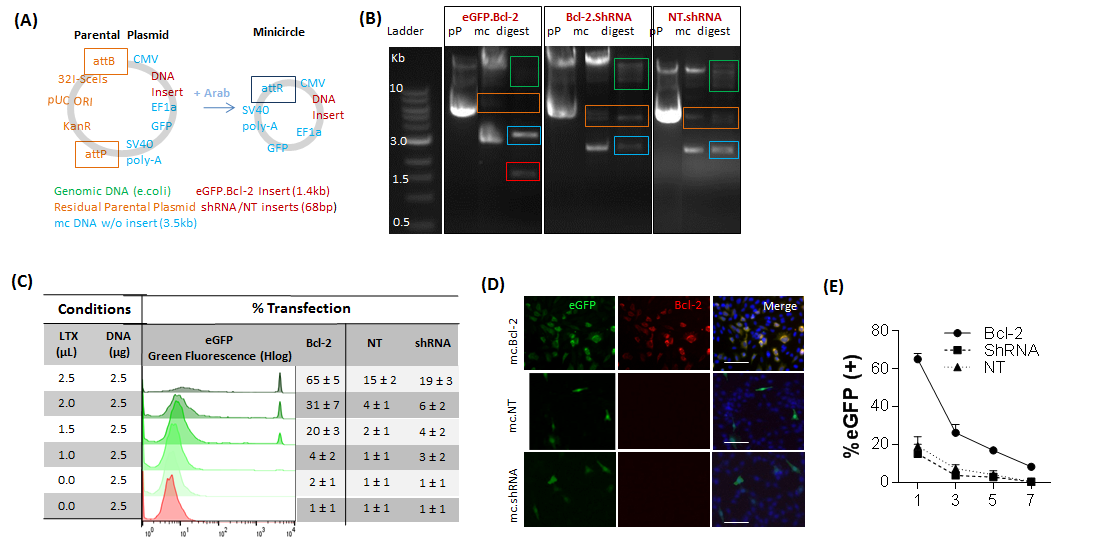
**

**Supplementary Figure 2:** Minicircle-driven *Bcl-2* expression in NSCs. a) Schematic showing minicircle construct generation after ligating 3 different inserts (eGFP.*Bcl-2*, *Bcl-2*.shRNA, and NT.shRNA) into the McEasy parental plasmid backbone. b) Image of agarose gel electrophoretesis verifying reduced size (~3kb) of each minicircle (mc) construct as compared to the parental plasmids (pP, ~8kb). Diagnostic restriction enzyme digests of each construct verify the presence of the 3kb minicircle backbone (blue boxes), and the 1.4kb *Bcl-2*.eGP insert (red box) though the 68pb shRNAs were too small to be visualized . The eGFP.*Bcl-2* minicircle construct was free of any residual parental plasmid (orange box) and genomic DNA (green box). c) NSCs were seeded at 90% confluency and allowed 24 hrs to recover. NSCs were then transfected at various Lipofectamine LTX concentrations (panel 1) for 24 hours before utilizing flow cytometry to the analyze transfection efficiency of all 3 constructs. Representative histograms for the mc.*Bcl-2* construct are shown (panel 2). d) Representative fluorescent images demonstrating positive eGFP expression in cells transfected (2.5 µL LTX) with minicircle constructs. *Bcl-2* co-localization only was observed in cells transfected with the eGFP.Bck-2 minicircle construct. Scale bar = 50 µm. e) At select time points transfected NSCs were monitored for eGFP expression using flow cytometry.

**
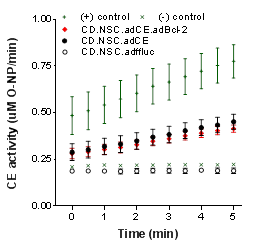
**

**Supplementary Figure 3: Co-transduction of Carboxyesterase and *Bcl-2* in NSCs.** a) NSCs were seeded at 50% confluency and allowed 48 hrs for CAR expression to recover. NSCs were then adenovirally transduced at MOI 20 using both Ad.*Bcl-2*, and ad.rCE for 24 hours in the presence of 1µg/mL protamine sulfate. CE enzyme activity was measured by conversion of *o*-nitrophenyl acetate substrate to *o*-nitrophenol and determined by spectrophotometry at 420 nm as previously described[28].


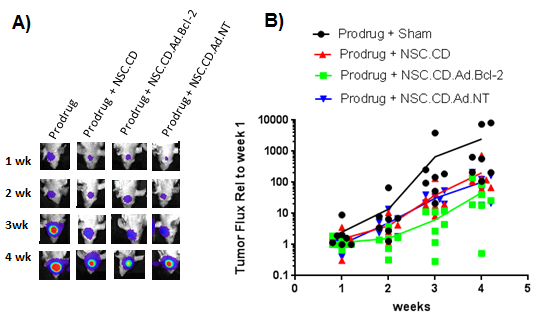


**Supplementary Figure 4: Therapeutic advantage of *Bcl-2* expression in NSCs in patient-derived glioma model.** a) Representative bioluminescence images of ffluc-expressing PBT-017 glioma cells after treatment with prodrug ± either parental or transduced NSC.CDs. b) Normalized bioluminescent tumor flux relative to week 1 in all mice (mean + individual data points shown).
